# Aquaporin 4-Mediated Glutamate-Induced Astrocyte Swelling Is Partially Mediated through Metabotropic Glutamate Receptor 5 Activation

**DOI:** 10.3389/fncel.2017.00116

**Published:** 2017-04-28

**Authors:** Zhongfang Shi, Wei Zhang, Yang Lu, Yi Lu, Lixin Xu, Qing Fang, Min Wu, Mei Jia, Yujiao Wang, Liping Dong, Xu Yan, Shaohua Yang, Fang Yuan

**Affiliations:** ^1^Department of Pathophysiology, Beijing Neurosurgical Institute, Beijing Tiantan Hospital, Capital Medical UniversityBeijing, China; ^2^China National Clinical Research Center for Neurological DiseasesBeijing, China; ^3^Beijing Key Laboratory of Central Nervous System InjuryBeijing, China; ^4^Center of Stroke, Beijing Institute for Brain DisordersBeijing, China; ^5^Beijing Key Laboratory of Translational Medicine for Cerebrovascular DiseaseBeijing, China; ^6^Department of Pharmacology and Neuroscience, University of North Texas Health Science CenterFort Worth, TX, USA

**Keywords:** astrocyte, swelling, glutamate, aquaporin 4, metabotropic glutamate receptor 5

## Abstract

Astrocytes are one of the most abundant cell types in the mammalian central nervous system (CNS), and astrocyte swelling is the primary event associated with brain edema. Glutamate, the principal excitatory amino acid neurotransmitter in the CNS, is released at high levels after brain injury including cerebral ischemia. This leads to astrocyte swelling, which we previously demonstrated is related to metabotropic glutamate receptor (mGluR) activation. Aquaporin 4 (AQP4), the predominant water channel in the brain, is expressed in astrocyte endfeet and plays an important role in brain edema following ischemia. Studies recently showed that mGluR5 is also expressed on astrocytes. Therefore, it is worth investigating whether AQP4 mediates the glutamate-induced swelling of astrocytes via mGluR5. In the present study, we found that 1 mM glutamate induced astrocyte swelling, quantified by the cell perimeter, but it had no effect on astrocyte viability measured by the cell counting kit-8 (CCK-8) and lactate dehydrogenase (LDH) assays. Quantitative reverse transcription polymerase chain reaction analyses revealed that AQP4, among AQP1, 4, 5, 9 and 11, was the main molecular expressed in cultured astrocytes. Glutamate-induced cell swelling was accompanied by a concentration-dependent change in AQP4 expression. Furthermore, RNAi technology revealed that AQP4 gene silencing inhibited glutamate-induced astrocyte swelling. Moreover, we found that mGluR5 expression was greatest among the mGluRs in cultured astrocytes and was co-expressed with AQP4. Activation of mGluR5 in cultured astrocytes using (S)-3,5-dihydroxyphenylglycine (DHPG), an mGluR5 agonist, mimicked the effect of glutamate. This effect was abolished by co-incubation with the mGluR5 antagonist fenobam but was not influenced by DL-threo-β-benzyloxyaspartic acid (DL-TBOA), a glutamate transporter inhibitor. Finally, experiments in a rat model of transient middle cerebral artery occlusion (tMCAO) revealed that co-expression of mGluR5 and AQP4 was increased in astrocyte endfeet around capillaries in the penumbra, and this was accompanied by brain edema. Collectively, these results suggest that glutamate induces cell swelling and alters AQP4 expression in astrocytes via mGluR5 activation, which may provide a novel approach for the treatment of edema following brain injury.

## Introduction

Brain edema is a common and fatal complication of many brain injuries, including ischemia, trauma, epilepsy, acute hepatic encephalopathy, severe hyponatremia (Lundgaard et al., 2014). The development of brain edema can cause an increase in intracranial pressure and brain herniation, ultimately leading to death. Unfortunately, for many years the standard treatment for brain edema has remained symptomatic and rather non-specific, owing to our incomplete understanding of the mechanisms underlying brain edema. Astrocytes are the main type of cell that support neurons and play important roles in physiological and pathological brain function, including regulating water and ion concentrations and providing nutrients and metabolites to neurons (Liu and Chopp, 2016). A large body of research indicates that astrocyte swelling is the primary event contributing to brain edema (Stobart and Anderson, 2013; Schitine et al., 2015; Wang and Parpura, 2016). Targeting astrocyte swelling may therefore be a novel strategy for treating this serious condition.

Glutamate, the primary excitatory neurotransmitter in the central nervous system (CNS), is a major contributor to the swelling of astrocytes. Glutamate plays crucial roles in brain development, brain maturation and synaptic plasticity (Kritis et al., 2015), with elevated levels of glutamate implicated in the etiology of a number of brain diseases including cerebral ischemia, epilepsy, as well as trauma, leading to astrocyte swelling (Stokum et al., 2015). Many studies have demonstrated that glutamate induces astrocyte swelling *in vivo* and *in vitro* (Yuan and Wang, 1996; Han et al., 2004; Willard and Koochekpour, 2013; Vella et al., 2015), likely via glutamate transporters and metabotropic glutamate receptors (mGluRs). Five types of glutamate transporter have been found in the mammalian CNS. The two types expressed predominantly in glial cells, glutamate transporter-1 (GLT-1) and glutamate and aspartate transporter (GLAST), maintain glutamate homeostasis under physiological and pathological conditions (Mogoanta et al., 2014). Several studies have shown that up-regulation of glutamate transporter reduces cerebral infarct volumes and astrocyte swelling (Verma et al., 2010; Benesova et al., 2012). mGluRs are G-protein coupled receptors, which are classified into three groups according to their pharmacological profiles and signal transduction pathways. These are groups I (mGluR1 and mGluR5), II (mGluR2 and mGluR3) and III (mGluR4, mGluR6, mGluR7 and mGluR8). In addition to being expressed in neurons, mGluRs are expressed in different types of glial cells, and their activation exerts a variety of effects that are essential for glial function under physiological and pathological conditions (D’Antoni et al., 2008; Wang and Zhuo, 2012). Unfortunately, it remains unclear which mGluR participates in glutamate-induced astrocyte swelling. The underlying molecular mechanisms are therefore worthy of further investigation.

The aquaporin (AQP) family is a class of water channel proteins involved in the regulation of water homeostasis (Verkman et al., 2014). To date, 13 members of the AQP family have been found to be conserved across mammalian species, 7 of which (AQP1, 3, 4, 5, 8, 9 and 11) are expressed in the CNS. Yamamoto et al. (2001) reported AQP 3, 4, 5, 8 and 9 expression in cultured rat astrocytes. AQP4, which is mainly expressed in astrocytes, is the most abundant water channel in the brain and plays an important role in water and ion homeostasis (Nielsen et al., 1997; Papadopoulos and Verkman, 2013). Altered AQP4 expression has been observed in a number of conditions including cerebral edema, neuromyelitis optica, epilepsy, brain tumor, Alzheimer’s disease, Parkinson’s disease, depression and drug addiction (Ribeiro Mde et al., 2006; Hirt et al., 2009; Zhao et al., 2012; Rajkowska and Stockmeier, 2013; Rajkowska et al., 2013; Di Benedetto et al., 2016). Several studies have also indicated that AQP4 plays roles in memory and synaptic plasticity through the regulation of glutamate transporter expression (Skucas et al., 2011; Li et al., 2012; Szu and Binder, 2016). Many studies showed that AQP4 took part in the formation and development of brain edema (Saadoun et al., 2002; Migliati et al., 2010; Tang et al., 2010; Jin et al., 2013; Akdemir et al., 2014; Katada et al., 2014; Rama Rao et al., 2014; Hirt et al., 2017), but it remains unclear if AQP4 is involved in glutamate-induced astrocyte swelling. Edema formation and cytotoxicity might be part of a vicious cycle, where cell swelling causes the release of cytotoxic compounds that leads to tissue damage and more swelling. An early stage intervention with AQP4 inhibitors would interfere with this vicious cycle by counteracting both the swelling and deleterious secondary events like ATP release and swelling-activated glutamate efflux. Gunnarson et al. (2008) recently found that the water permeability of astrocytes is altered by group I mGluRs, and the molecular target for this phenomenon is the serine 111 residue of AQP4. Furthermore, Illarionova et al. (2010) showed that mGluR5, AQP4 and Na^+^/K^+^-ATPase form a tripartite macromolecular complex in the astrocyte plasma membrane. Therefore, we speculated that AQP4 mediates glutamate-induced astrocyte swelling via mGluR5 activation. In the present study, we measured glutamate-induced astrocyte swelling and changes in AQP4 expression. We then investigated how AQP4 RNAi or pretreatment with mGluR5 agonists or antagonists and a GLT inhibitor affects glutamate-induced astrocyte swelling and AQP4 expression levels. Finally, we evaluated AQP4 and mGluR5 expression after cerebral ischemia in a rat model of transient middle cerebral artery occlusion (tMCAO).

## Materials and Methods

### Animals

Male Sprague-Dawley (SD) rats (280–320 g) and neonatal Wistar rats were purchased from the Institute of Laboratory Animal Science, affiliated with the Chinese Academy of Medical Sciences (Certificate No, SCSK (Beijing) 2005–0013).

Although our previous study showed that astrocytes from Wistar rats are more susceptible to glutamate-induced swelling than that from SD rats (Shi et al., 2016), other studies displayed that SD rats had a relatively large infarcted volume and low coefficient of variation compared with Wistar rats after focal cerebral ischemia (Duverger and MacKenzie, 1988; Bardutzky et al., 2005). In the current study, we carried out the model of tMCAO in SD rats.

SD rats were housed under a 12:12-h light/dark cycle at a room temperature of 24 ± 1°C and relative humidity of 55%–65%, with free access to food and water.

All animal procedures were conducted according to the Guidelines for the Care and Use of Laboratory Animals, and were approved by the Animal Care and Use Committee at Beijing Neurosurgical Institute (No. 201401007).

### Cell Culture

Astrocytes were prepared from cerebral cortices of Wistar rats within 1 day of birth, according to the method described by McCarthy and de Vellis (1980). Briefly, cortical cells were dissociated into single cells that were incubated in modified Eagle’s medium (MEM, Gibco, Grand Island, NY, USA) supplemented with 10% fetal bovine serum (FBS, Gibco), and maintained at 37°C in a 5% CO_2_ incubator. The medium was renewed every 3–4 days. The purity of the cell culture was assessed by immunofluorescence staining for anti-glial fibrillary acidic protein (GFAP) antibody (Dako, Glostrup, Denmark). Subculture to obtain more astrocytes was performed when the astrocytes were confluent, on the 10th day of primary culture, and these second-passage astrocytes were used in all experiments. Individual experimental astrocyte cultures were exposed to different concentrations of glutamate (0, 1 and 10 mM) for 48 h, and 1 mM glutamate for 0, 1, 3, 6, 12, 24, or 48 h. In the control group, only the MEM solution was replaced. The selective mGluR5 agonist (S)-3,5-dihydroxyphenylglycine (DHPG, 100 μM, TOCRIS, Bristol, UK), the selective mGlu5 antagonist fenobam (300 μM, TOCRIS) and the non-selective glutamate transporter inhibitor DL-threo-β-benzyloxyaspartic acid (DL-TBOA, 1 mM, TOCRIS) were added for 48 h with or without 1 mM glutamate to investigate their effect on AQP4 expression and astrocyte swelling.

### Immunocytochemistry and Quantification

Astrocytes cultured on glass coverslips were fixed in acetone for 10 min. After being blocked with goat serum in phosphate-buffered saline (PBS) for 20 min, cells were sequentially incubated with rabbit polyclonal antibodies against AQP4 (1:30, Abcam, Cambridge, UK) and GFAP (1:50, DAKO) at 4°C overnight, and Alexa Fluor 488 Goat Anti-Rabbit IgG (H+L) antibody (1:200, Life Technologies, Carlsbad, CA, USA) for 1 h at room temperature, as described previously (Shi et al., 2015). Finally, the cells were examined under an inverted Zeiss Axio Observer fluorescence microscope (Carl Zeiss, Oberkochen, Germany). The control group was treated with PBS instead of the primary antibody.

Double-immunofluorescent staining for AQP4 and mGluR5 was carried out on cultured astrocytes. The experimental protocol was as follows: cells were incubated with rabbit polyclonal antibody against AQP4 (1:30, Abcam) and mouse polyclonal antibody against mGluR5 (1:50, Abcam) overnight at 4°C. This was followed by incubation with secondary antibodies (TRITC/Alexa Fluor 488-conjugated anti-rabbit/mouse IgG) for 1 h at room temperature.

Immunofluorescent staining for AQP4 and GFAP were analyzed using ImageJ software (National Institutes of Health, Bethesda, MD, USA; Schneider et al., 2012; Rajkowska et al., 2013). Images were randomly acquired in all zones from three samples per condition in three independent experiments. Three images were acquired using at least three slides per one sample. The images underwent black and white reverse processing, and the average optical density (AOD) was calculated in every image. Quantitative analysis of the colocalization of AQP4 and GluR5 after tMCAO was performed using the colocalization function of ImageJ software. The extent of colocalization was determined by calculating the Mander’s overlap coefficient (MOC; R) which indicates an actual overlap of the signals and is considered to represent the true degree of colocalization. The values of MOC are in the range of 0–1.0. If the image has an overlap coefficient of 1, it implies that 100% of both selected channels overlap. A value of zero means that there are no overlapping pixels (Zinchuk and Grossenbacher-Zinchuk, 2009). The data were obtained from three independent fields containing more than three randomly selected images per condition in three different experiments.

### Measurement of the Cell Perimeter

Our previous study has showed that glutamate at 1 mM caused a significant increase in astrocytic volume by measurement of [^3^H]-O-methyl-D-glucose uptake (Yuan and Wang, 1996). Sorensen et al. (2001) demonstrated that changes in volume can be detected accurately with the perimeter method, and we used the perimeter to represent astrocyte volume. These values were calculated after GFAP immunofluorescence staining using the Image Pro Plus software package (Media Cybernetics, Rockville, MD, USA). Ten cells in each field under high magnification (200×) were randomly selected. Three fields in three wells were measured in every group, and the average values of nine fields were calculated as the cell perimeter of each group.

### Cell Viability Assays

Cell viability of astrocytes was evaluated using the cell counting kit-8 (CCK-8) and lactate dehydrogenase (LDH) assays (He et al., 2015).

For the CCK-8 assay, cells were pretreated with glutamate at the concentrations and durations described above according to their experimental group. After adding 10 μl CCK-8, the plates were incubated for 2 h at 37°C. The optical density (OD) was detected at a wavelength of 450 nm on Multimode Plate Readers (Tecan, Mannedorf, Switzerland). The OD values for the experimental groups are represented as the percentage change compared with control.

To assess plasma membrane integrity after incubation with glutamate, the activity of LDH released into the medium was measured using a detection kit (Applygen Technologies, Beijing, China). Levels of LDH leakage were calculated according to the manufacturer’s instructions using a microplate recorder at 440 nm (OD440). Values for LDH leakages are expressed as extracellular LDH/total extracellular and intracellular LDH (%). All experiments were replicated three times with three samples for each condition in each experiment.

### Aquaporin 4 Knockdown by siRNA

Cells were seeded into a 24-well plate for 24 h before transfection to the complete medium. As previously described, RNA duplexes of 21 nucleotides specific for rat AQP4 sequences were synthesized, together with a control siRNA (AQP4 siRNA mismatch sequence; Qi et al., 2011). The AQP4 sense sequence was 5′-aagaucagcaucgccaagucu-3′ (RiboBio, Guangzhou, China). Transfection of siRNA duplexes was carried out with the Lipofectamine RNAiMax transfection reagent according to the manufacturer’s instructions (Invitrogen, Carlsbad, CA, USA; 3 ml/L), and the mixture was added to the cultured astrocytes at a final concentration of 30 nM for 48 h (Zhang et al., 2014). To determine transfection efficiency, the expression levels of AQP4 mRNA and protein were measured using Western blot, quantitative reverse transcription PCR (qRT-PCR) and immunofluorescence staining.

### Real-Time Quantitative Reverse Transcription PCR (qRT-PCR) and Reverse Transcription PCR (RT-PCR)

Total RNA was isolated using the TRIzol regent (Invitrogen, Carlsbad, CA, USA), according to the manufacturer’s instructions. Complementary DNAs (cDNAs) were prepared from 1 μg total RNA using the Reverse Transcription System (Promega, Madison, WI, USA).

Expression levels of AQPs and AQP4 mRNA in cultured astrocytes were measured by qRT-PCR using a LightCycler 480 (Roche, Basel, Switzerland). All of the primer sequences used in this experiment are shown in Table 1. All samples, along with the housekeeping gene β-actin, were analyzed using qRT-PCR. Conditions for PCR were as follows: 95°C for 10 min; followed by 40 cycles of 95°C for 15 s, 58°C for 30 s, and 72°C for 30 s; and a final extension at 72°C for 10 min. The specificity of the measured signal was determined using a dissociation curve, consisting of a single peak. The comparative Ct method (2^−∆∆^Ct) was used for quantification (Balci et al., 2016). Results reported are expressed as the AQP4/β-actin ratio. All experiments were replicated three times with three samples for each condition in three independent cultures.

**Table 1:** **Primer sequences of the aquaporin (AQP) family**.

Gene	Primer sequence (5′ to 3′),
AQP0	F: 5′-AGATGTCCTTGCTCCGTGCTT-3′
	R: 5′-CGTGTTGAGTGCTAGGTTTCC-3′
AQP1	F: 5′-TCCGGGCTGTCATGTATATCATC-3′
	R: 5′-ATGGCGGAGGCAACGA-3′
AQP2	F: 5′-GGCCACCTCCTTGGGATCTA-3′
	R: 5′-GGAGCGGGCTGGATTCA-3′
AQP3	F: 5′-TGGGATTGTTTTTGGGCTCTA-3′
	R: 5′-AACAAGCTCATTGCCAGCAA-3′
AQP4	F: 5′-TGAATCCAGCTCGATCCTTTG-3′
	R: 5′-TATCCAGTGGTTTTCCCAGTTTC-3′
AQP5	F: 5′-CACCCTCATCTTCGTCTTCTTTG-3′
	R: 5′-CAGAGCCGAGGGCCACTT-3′
AQP6	F: 5′-GGCCCTGCGGTCATTG-3′
	R: 5′-CGGTCCTACCCAGAAGATCCA-3′
AQP7	F: 5′-GATGGTGTTTGGCCTTGGTT-3′
	R: 5′-GCTGCCAAGCCTTTCTCCTA-3′
AQP8	F: 5′-TGGGTGCCGTCAATGAGA-3′
	R: 5′-ACAGAGAAACCAATGGAGAATGG-3′
AQP9	F: 5′-GCATCGGCAGTCGTGATG-3′
	R: 5′-TGATGTGGCCCCCAGAGAT-3′
AQP11	F: 5′-TCACAGGAGCGCTGTTTAACC-3′
	R: 5′-CGTCAAAGCACGGGAAGTG-3′
AQP12	F: 5′-TGTCACCTTGCTCCTTGTAGAACCC-3′
	R: 5′-TAGATGCCCTCCTAGCCACCTCA-3′
β-actin	F: 5′-CTTCAACACCCCAGCCATGT-3′
	R: 5′-ACCAGAGGCATACAGGGACAA-3′

Expression of mGluR was detected by RT-PCR using gene-specific primers (Table 2). Reactions were performed in a Mini-Cycler thermal cycler (Life Technologies). All samples were denatured at 94°C for 5 min and amplified over 40 cycles using the following amplification parameters: 94°C for 30 s, 60°C for 30 s, and 72°C for 45 s. All RT-PCR experiments contained negative controls, from which template cDNA was omitted. The RT-PCR product was electrophoresed on a 2% agarose gel containing ethidium bromide. The gel bands were visualized in a multifunctional gel imaging system (Bio-Rad, Hercules, CA, USA). The relative amount of mGluR was expressed as OD relative to that of β-actin.

**Table 2:** **Primer sequences of metabotropic glutamate receptors (mGluRs)**.

Gene	Primer sequence (5′ to 3′)
mGluR1	F: 5′-CATTCCTGTGATTCCCTGTGTTT-3′
	R: 5′-TCCGACTGGTTGATAGTATTTGCT-3′
mGluR2	F: 5′-AGTCTTCCACTGTTCATTCTGCTAA-3′
	R: 5′-TGTTATTGATGGTTCCTTTTTGC-3′
mGluR3	F: 5′-GGTGTCCGTGTGGCTTATCC-3′
	R:5′-CATTGTTGTCGTCTGCACTCTGTAG-3′
mGluR4	F: 5′-CTTGGTGCTGAGGACAGTAGAGAC-3′
	R : 5′-GAGCAGAATGGAAAAATATGGAAG-3′
mGluR5	F: 5′-GCCTTCGTGCCTATCTACTTTGG-3′
	R: 5′-CCGGGTACTCTTCTCATTCTGG-3′
mGluR6	F: 5′-TGCCCTTTCTCTGCTTCTCCT-3′
	R: 5′-CTATGGTTTTGAATGCCTGCC-3′
mGluR7	F: 5′-GTGTCTTCATTTGGTTTGGGGT-3′
	R: 5′-TCATGGAGATTGTAAGCGTGGT-3′
mGluR8	F: 5′-ATCAGAGACCAAACATCAACCG
	R: 5′-TGAAACACATACCAAGTCCCAAG
β-actin	F: 5′-CGTTGACATCCGTAAAGACC-3′
	R: 5′-CTAGGAGCCAGAGCAGTAATC-3′

### Transient Focal Cerebral Ischemia and Double-Immunofluorescent Staining

TMCAO in rats was carried out as previously described (Fang et al., 2016). Rats were subjected to 1 h ischemia followed by 48 h of reperfusion. They were then sacrificed, and the brains were harvested. An approximately 4-mm-thick piece of brain tissue in the percussion area was fixed in 4% paraformaldehyde at 4°C for 24 h and embedded in paraffin, and then sliced into 4-μm sections. Hematoxylin and eosin (HE) staining were performed after dewaxing and hydration.

Sections were blocked in 5% bovine serum albumin after fixing, followed by incubation with primary antibodies (AQP4 1:200, GFAP 1:100, mGluR5 1:100) overnight at 4°C. This was followed by incubation with secondary antibodies (TRITC/Alexa Fluor 488-conjugated anti-rabbit/mouse IgG) for 1 h at room temperature. Sections were covered with DAPI fluorescence mounting medium after washing with PBS. For the negative control, the primary antibodies were omitted. The sections were photographed with a fluorescence microscope (Carl Zeiss).

### Western Blot

AQP4 protein expression was determined by Western blot as described in our previous publication (Shi et al., 2015). The astrocytes were added to 0.25% trypsin for 10 min at room temperature and collected with a cell scraper. The harvested astrocytes were lysed in cell lysis buffer on ice, and the cells were disrupted with an ultrasonic instrument. Protein concentrations were determined by the BCA method. Protein samples were denatured at 85°C for 15 min followed by sodium dodecyl sulfate polyacrylamide gel electrophoresis (SDS-PAGE) on 10% polyacrylamide gels. The gels were blotted onto 0.45-μm polyvinylidene fluoride (PVDF) membranes at 22 V for 40 min. Non-specific binding sites were blocked with 5% nonfat milk in Tris-saline buffer (TBS) for 2 h at room temperature. The PVDF membranes were incubated with polyclonal antibodies against AQP4 (rabbit anti-rat, 1:500, Millipore, Billerica, MA, USA) overnight at 4°C. Subsequently, the PVDF membranes were incubated with horseradish peroxidase-conjugated goat anti-rabbit IgG (1:1000, Cell Signaling Technology, Danvers, MA, USA) at room temperature for 1 h. The specific bands were detected using an enhanced chemiluminescence reagent kit according to the manufacturer’s instructions. β-actin (mouse anti-rat, 1:5000, Sigma, St. Louis, MO, USA) was used as a loading control. The bands were imaged with the ChemiDoc MP (Bio-Rad) and analyzed using the software (Bio-Rad). All results are based on nine independent samples that were repeated three times. AQP4 protein expression levels were normalized to β-actin.

### Statistical Analysis

Statistical analyses were performed using SPSS 11.5 for Windows (SPSS Inc., Chicago, IL, USA). All data are expressed as the mean ± SD, and obtained from three independent samples per condition in three independent experiments (*n* = 9). One-way analysis of variance (ANOVAs) followed by Honestly Significant Difference (HSD) *post hoc* tests were used to statistically analyze CCK8 assay, LDH leakage, cell perimeter, and AQP4 mRNA and protein expression data. We performed *t*-tests to analyze double immunofluorescent staining for AQP4, GFAP and mGluR5 expression. A *P*-value < 0.05 was considered statistically significant.

## Results

### Glutamate Induces Cell Swelling of Cultured Rat Astrocytes

**Figure 1:**
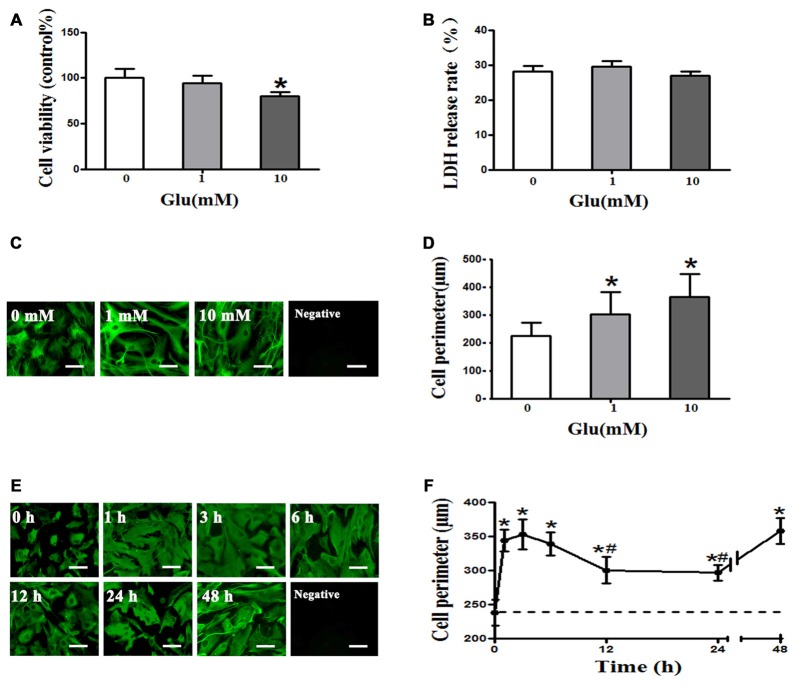
**Glutamate-induced cell swelling in cultured astrocytes. (A)** Measurement of astrocyte survival after treatment with 0, 1, or 10 mM glutamate for 48 h by cell counting kit-8 (CCK-8) assay showed that 1 mM glutamate had no effect on astrocytes viability, whereas 10 mM glutamate decreased astrocyte viability.** (B)** Lactate dehydrogenase (LDH) release rates measured using an LDH kit showed that 1 and 10 mM glutamate had no effect on astrocyte viability. **(C,E)** Immunofluorescence staining for GFAP (shown in green). Representative images showing the effects of exposure of astrocytes to 0, 1, or 10 mM glutamate for 48 h **(C)**, and 1 mM glutamate for 0, 1, 3, 6, 12, 24, or 48 h **(E)**. Scale bar shows 200 μm.** (D)** Quantitative analysis of image data from panels in **(C)** showed that astrocyte cell perimeter significantly increased after exposure to 1 or 10 mM glutamate for 48 h. **(F)** Quantitative analysis of image data from panels in **(E)** showed that astrocytes swelled 1 h after treatment with 1 mM glutamate and maintained a similar level of swelling at 3 and 6 h. Astrocytes swelling then decreased at 12 and 24 h but again increased at 48 h compared to the control group (the dotted line). Control group corresponds to “0” in **(A), (B), (D), (F)**. *P < 0.05, compared with control; ^#^*P* < 0.05, compared with 1 h; *n* = 9; graphed data show mean ± SD.

The CCK8 assay showed that 1 mM glutamate had no effect on astrocyte viability, whereas viability was significantly decreased by 10 mM glutamate (*P* < 0.05, Figure 1A). However, the LDH release rate indicated that 1 and 10 mM glutamate had no effect on astrocyte viability (*P* > 0.05, Figure 1B). Cell perimeter measurements showed 1 or 10 mM glutamate after 48 h incubation induced astrocyte swelling ((225.18 ± 46.87) μm, (302.41 + 79.17) μm, (363.86 + 83.92) μm, *P* < 0.05, Figures 1C,D), indicating that glutamate induce astrocyte swelling in a concentration-dependent manner. Moreover, 1 mM glutamate-induced astrocyte cell swelling at 1 h ((344.03 ± 15.98) μm) that was maintained at 3 h ((353.2 ± 22.39) μm) and 6 h ((339.14 ± 17.16) μm), compared to the control group ((237.85 ± 18.91) μm). The rate of astrocyte swelling lessened at 12 h ((300.52 ± 19.46) μm) and 24 h ((296.65 ± 11.83) μm), but the degree of swelling was still higher than that of the control group ((237.85 ± 18.91) μm). The level of astrocyte swelling again increased at 48 h ((358.29 ± 18.64) μm; *P* < 0.05, Figures 1E,F). These results suggest that 1 mM glutamate induced cell swelling but had no effect on cell viability in cultured rat astrocytes.

### Glutamate-Induced Astrocyte Swelling Is Mediated by AQP4

**Figure 2:**
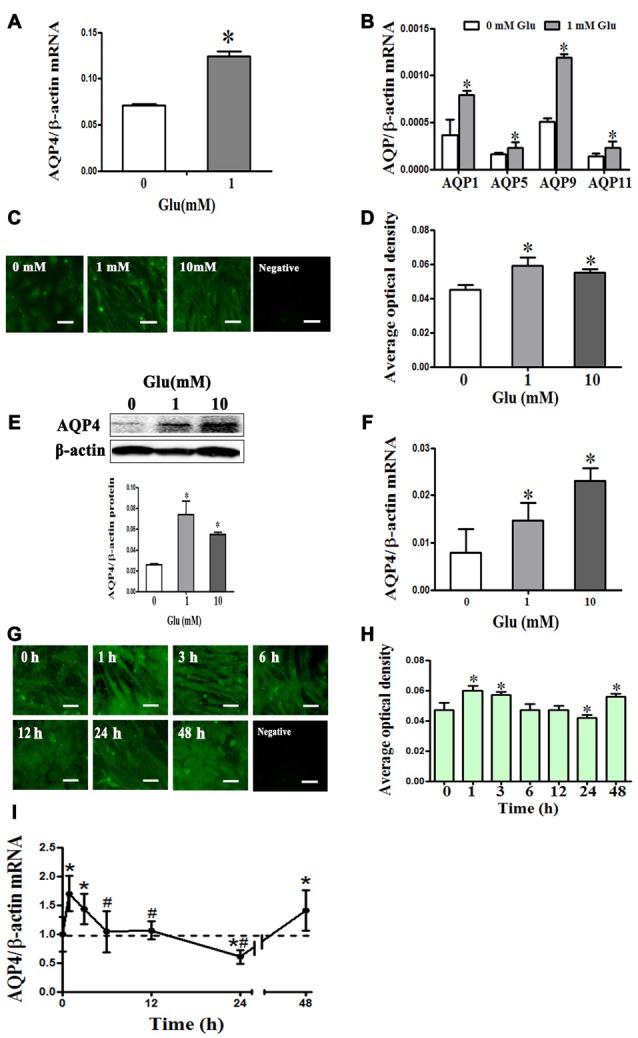
**Glutamate-induced changes in aquaporin 4 (AQP4) expression in cultured astrocytes. (A,B)** Change in expression AQP family subtype mRNAs after incubation with 1 mM glutamate for 48 h. Results showed that AQP4 expression was the highest, followed by AQP11. Besides AQP4 and AQP11, the expression of AQP1, 5, and 9 mRNAs were also found in cultured astrocytes. After incubation in 1 mM glutamate for 48 h, the expression of AQP1, 4, 5, 9 and 11 were all significantly increased approximately two-fold compared to levels before incubation. **(C,G)** Immunofluorescence staining for AQP4 (shown in green). Representative images showing effects of 0, 1, or 10 mM glutamate for 48 h **(C)**, and exposure to 1 mM glutamate for 1, 3, 6, 12, 24, or 48 h **(G)**. Scale bar shows 200 μm. **(D–H)** Quantitative analysis of data from **(C)** and **(G)**. **(E,F)** Levels of AQP4 protein and mRNA after 48-h glutamate incubation were by detected Western blot and quantitative reversetranscription-PCR (qRT-PCR), respectively. Compared with the control group, 1 or 10 mM glutamate both induced an increase in AQP4 expression. **(I)** AQP4 mRNA expression was measured by qRT-PCR. Results showed that AQP4 mRNA significantly increased at 1 h after treatment with 1 mM glutamate, and this level was maintained at 3 h. AQP4 expression was reduced to a normal level at 6 and 12 h, before falling significantly below the normal level at 24 h. However, expression of AQP4 mRNA again increased at 48 h. Control group corresponds to “0” in **(D), (E), (F), (H), (I)**. *P < 0.05, compared with control; ^#^*P* < 0.05, compared with 1 h; *n* = 9; graphed data show mean ± SD.

We measured the expression profile of AQP family mRNA in cultured rat astrocytes by qRT-PCR. The results revealed that AQP4 expression was the highest, followed by AQP11. However, it is worth noting that the level of AQP4 was 100 times higher than that of AQP11. Besides AQP4 and AQP11, we also observed AQP1, 5,and 9 mRNA expressions in cultured astrocytes. After incubation in 1 mM glutamate for 48 h, expression of AQP1, 4, 5, 9 and 11 were significantly increased by approximately two-fold compared to levels before incubation (*P* < 0.05, Figures 2A,B). These results suggest that AQP4 is the main molecule in astrocytes that mediates water homeostasis in normal and pathophysiological conditions.

To explore how AQP4 levels in astrocytes change after incubation with 1 mM glutamate, we performed immunofluorescence staining, Western blot and qRT-PCR. After 48-h incubation with glutamate (1 and 10 mM), compared to the control group (0.026 ± 0.003), AQP4 protein expression detected by Western blot was increased ((0.074 ± 0.013), (0.055 ± 0.004), respectively, *P* < 0.05, Figure 2E), and which were consistent with those from AQP4 immunofluorescence staining (Figures 2C,D); AQP4 mRNA expression was also increased ((0.007885 ± 0.005019), (0.014715 ± 0.0036), (0.0231 ± 0.003), respectively, *P* < 0.05, Figure 2F), indicating that glutamate increased AQP4 expression in concentration-dependent manner.

Meanwhile, we observed that following incubation with 1 mM glutamate, compared to the control group (0.995 ± 0.298), AQP4 expression was significantly increased at 1 h (1.6990 ± 0.308), maintained at 3 h (1.4320 ± 0.260) and reduced to normal levels at 6 h (1.0410 ± 0.358) and 12 h (1.064 ± 0.157). At 24 h, levels fell significantly below the normal level (0.6070 ± 0.118). However, AQP4 mRNA expression again increased at 48 h (1.4110 ± 0.350; *P* < 0.05, Figure 2I). AQP4 immunofluorescence staining yielded results that corroborated the changes observed in AQP4 mRNA expression (Figures 2G,H). These results indicate that glutamate induced dynamic changes in AQP4 expression in cultured rat astrocytes.

**Figure 3:**
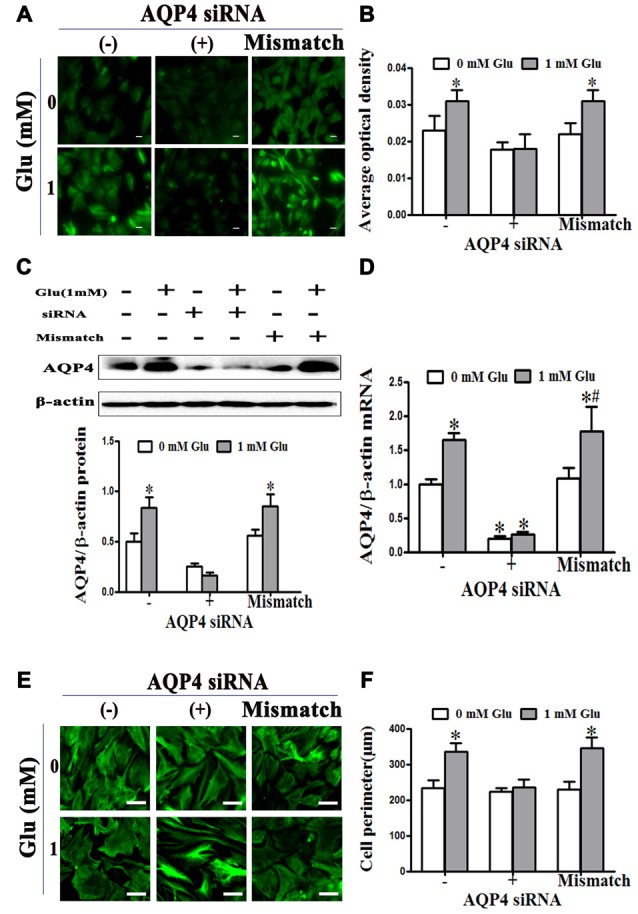
**Glutamate-induced swelling of astrocytes was mediated by AQP4. (A,C)** Levels of AQP4 protein in cultured astrocytes were measured by immunofluorescence and western blot, respectively, after incubation with glutamate and AQP4 siRNAs. In the images, AQP4 is shown in green **(A)**. Scale bar shows 200 μm. **(B)** Quantitative analysis of data from **(A)**. The results showed that 1 mM glutamate increased the level of AQP4 protein. This was inhibited by AQP4 siRNA transfection, but not the mismatch siRNA transfection. **(D)** Expression of AQP4 mRNA was detected by qRT-PCR. The result was consistent with that of AQP4 protein expression. **(E)** Astrocytes were transfected with siRNA for 24 h, before exposure to 1 mM glutamate for 48 h. Representative photos taken with an inverted fluorescence microscope show GFAP immunofluorescence in green. Scale bar shows 200 μm. **(F)** Quantitative analysis of cell perimeter from images in **(E)**. One millimolar glutamate caused an increase in astrocyte perimeter which was blocked when AQP4 siRNA was transfected into astrocytes to silence the gene. In contrast, mismatch siRNA transfection failed to inhibit the increase of astrocyte perimeter induced by glutamate. *P < 0.05, compared with AQP4 siRNA (−), 0 mM glutamate; ^#^*P* < 0.05, compared with mismatch; *n* = 9; graphed data show mean ± SD.

We further investigated the effect of AQP4 RNAi on glutamate-induced astrocyte swelling. The results showed that 1 mM glutamate increased the level of AQP4, which was inhibited by transfection with AQP4 siRNA for 24 h before glutamate treatment, but not mismatch siRNA (*P* < 0.05, Figures 3A–D). Moreover, we found that 1 mM glutamate caused an increase in astrocyte volume that was blocked when AQP4 siRNA was transfected into astrocytes to silence the gene. In contrast, mismatch siRNA transfection failed to inhibit the increase of astrocyte volume induced by glutamate (*P* < 0.05, Figures 3E,F). These results indicate that AQP4 is the molecular target that mediates glutamate-induced astrocyte swelling.

### Metabotropic Glutamate Receptor 5 Mediates Glutamate-Induced Astrocyte Swelling and Changes in AQP4 Expression

**Figure 4:**
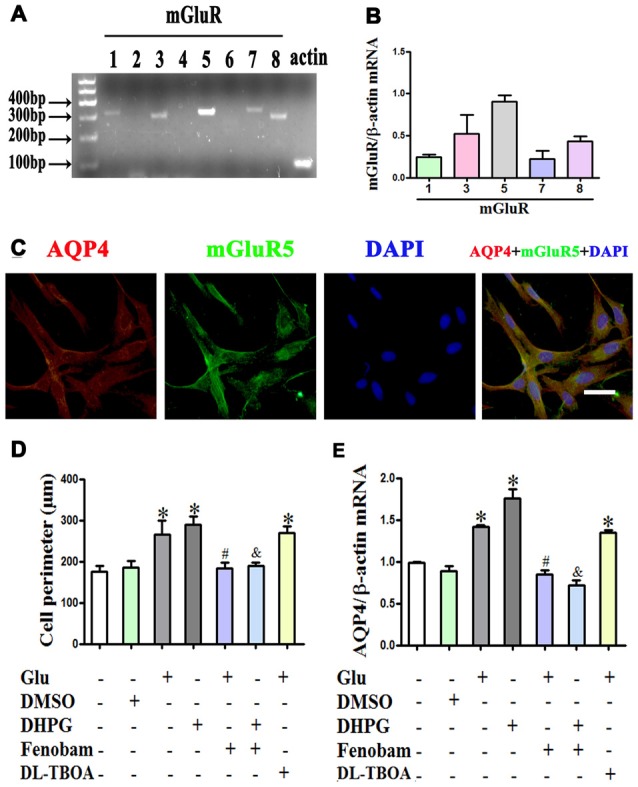
**mGlu5 mediated glutamate-induced astrocyte swelling and changes in AQP4 expression. (A)** Expression of metabotropic glutamate receptor (mGluR) in cultured astrocytes, as measured by RT-PCR. **(B)** Quantitative analysis of data from **(A)**. Results showed that mGluR1, 3, 5, 7 and 8 were expressed in astrocytes, and mGluR5 expression was the highest. **(C)** Co-expression of AQP4 and mGluR5 in cultured astrocytes, as measured by double-immunofluorescence staining. **(D)** Quantitative analysis of cell perimeters after GFAP immunofluorescence staining. Compared to the control, the cell perimeters of astrocytes in the Glu and DHPG groups were larger, whereas the DMSO group showed no difference compared to the control. Cell perimeters in the Glu + Fenobam group were smaller than in the Glu group, and likewise smaller in the DHPG + Fenobam group vs. the DHPG group. However, there were no cell perimeter differences between the Glu + DL-TBOA and Glu groups.** (E)** Quantitative analysis of qRT-PCR results showed that AQP4 mRNA expression in the Glu and DHPG group was higher than in the control group, whereas the DMSO group did not differ from control. Compared to the Glu group, expression was lower in the Glu + Fenobam group, but the level in the Glu + DL-TBOA group did not differ. The level of AQP4 mRNA was lower in the DHPG + Fenobam group compared to the DHPG group. *P < 0.05, compared to the control group; ^#^*P* < 0.05, compared to the Glu group; ^&^*P* < 0.05, compared to the DHPG group; *n* = 9; graphed data show mean ± SD. DHPG, (S)-3,5-dihydroxyphenylglycine, an mGluR5 agonist; DL-TBOA, DL-threo-β-benzyloxyaspartic acid, a glutamate transporter-1 (GLT-1) inhibitor.

Given that mGluR activation might account for the effect of glutamate on astrocyte swelling (Yuan and Wang, 1996; Gunnarson et al., 2008), we investigated the expression profile of mGluRs using RT-PCR. We found that mGluR1, 3, 5, 7 and 8 were expressed in astrocytes, with mGluR5 expression the highest and mGluR3 the second highest (Figures 4A,B). Immunofluorescence double staining showed the co-expression of AQP4 and mGluR5 in cultured astrocytes (Figure 4C).

Astrocytes were treated with 1 mM glutamate or 100 μM DHPG (a specific mGluR5 agonist), alone or with 300 μM fenobam (a selective mGluR5 antagonist) for 48 h. The results showed that DHPG induced astrocyte swelling and increased AQP4 expression, similar to the effect of glutamate. In contrast, fenobam reversed DHPG-induced cell swelling and increased AQP4 expression in cultured astrocytes (*P* < 0.05, Figures 4D,E). Furthermore, pretreatment with 1 mM DL-TOBA, a glutamate transporter inhibitor, did not abolish cell swelling or changes in AQP4 expression induced by glutamate or DHPG (Figures 4D,E). These results indicate that changes in AQP4 expression mediating glutamate-induced astrocyte swelling are directly dependent on mGluR5 activation.

### Co-Expression of mGluR5 and AQP4 Are Increased in Ischemic Brain Edema

**Figure 5:**
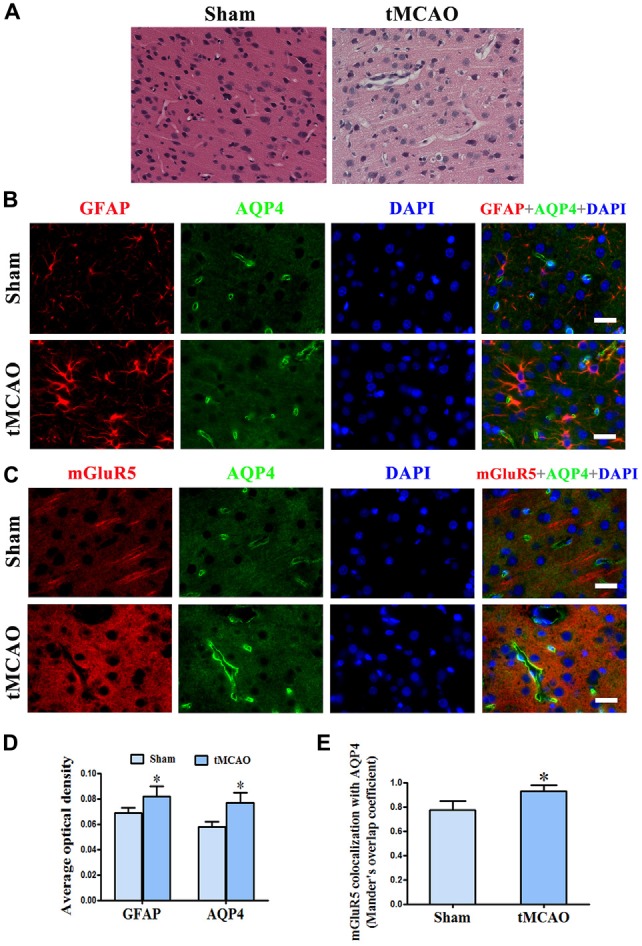
**Increased co-expression of mGluR5 and AQP4 in a rat model of transient middle cerebral artery occlusion (tMCAO). (A)** Hematoxylin and eosin (HE) staining showed that neuronal cells in the sham group were arranged regularly, and glial cells and capillary morphogenesis were normal. After tMCAO, most cell arrangement was disordered, with pyknotic or severely shrunken nuclei; in certain areas, hypervacuolization and cellular edema were found. **(B)** Double immunofluorescence staining showed the expression of GFAP (red) and AQP4 (green) in astrocytes, which increased after tMCAO. **(C)** Double immunofluorescence staining showed that mGluR5 (red) and AQP4 (green) were rarely co-expressed in pericapillary areas in the normal brain, but co-expression was increased in the pericapillary area and cell membranes of astrocytes after tMCAO. Scale bar shows 20 μm. **(D)** Statistical analysis of chart **(B)**.** (E)** Colocalization of AQP4 and mGluR5 was determined using ImageJ software. Three independent fields containing more than three randomly selected images per condition in three different experiments were assessed and analyzed, and the average Mander’s overlap coefficient was calculated. *P < 0.05, compared to the sham group, graphed data show mean ± SD.

We further studied the relationship between mGluR5 and AQP4 after brain edema using a rat model of tMCAO. Staining with HE showed regular neuronal cells arrangement and normal glial cells and capillary morphogenesis in the sham group. After tMCAO, most cells were arranged disorderly, with pyknotic or severely shrunken nuclei; in certain areas, hypervacuolization and cellular edema were found (Figure 5A). GFAP and AQP4 double immunofluorescence staining showed that AQP4 in astrocytes was increased after tMCAO (Figures 5B,D). The quantitative analysis revealed that the colocalization of mGluR5 and AQP4 measured by MOC was increase in sham group (0.7740 ± 0.0326) compared with that in tMCAO group (0.9300 ± 0.0212; Figure 5E). Co-expression of mGluR5 and AQP4 was rare in the pericapillary area of astrocytes in normal brain tissue, but it increased after tMCAO (Figures 5C,E). Increased co-expression was also found in the cell membranes of astrocytes following tMCAO, suggesting that ischemic cerebral edema might influence mGluR5 regulation of AQP4.

## Discussion

Previous studies have demonstrated that glutamate induces astrocyte swelling and that AQP4 plays a critical role in brain edema formation and resolution, but little is known regarding the role of AQP4 in glutamate-induced astrocyte cell swelling and the underlying mechanisms. The present study provides the first evidence that AQP4 mediates glutamate-induced astrocyte swelling and that mGluR5 activation is involved in AQP4-mediated astrocyte swelling induced by glutamate.

Glutamate is the primary excitatory neurotransmitter of the CNS, and elevated glutamate can lead to neuronal death and astrocyte swelling. In this study, we found that stimulation of cultured astrocytes with 1 mM glutamate increased cell swelling but had no effect on cell viability. Consistent with our results, many studies have demonstrated that swelling of cultured astrocytes occurs following glutamate treatment (Hansson et al., 1994; Yuan and Wang, 1996; Gunnarson et al., 2008). In contrast, Zhang et al. (2014) reported that 1 mM glutamate induced neuronal cell death in culture (Zhang and Bhavnani, 2006), while Szydlowska et al. (2006) found apoptotic alterations in the nuclear morphology of cultured astrocytes after 24-h incubation in 50–100 mM glutamate. Chan et al. (1990) showed that incubation with glutamate at concentrations hazardous to neurons induces astrocyte swelling *in vitro*, suggesting that astrocytes are more susceptive to swelling than neurons. The mechanisms underlying glutamate-induced astrocyte swelling are very complex and might relate to AQP4 expression in astrocyte endfeet.

Accumulating evidence suggests that AQP4 is the most important AQP in the brain and plays a key role in the development of brain edema. In the present study, we found that AQP4 mediated glutamate-induced astrocyte swelling, which may provide new therapeutic targets for brain edema therapy. In our experiment, we observed that glutamate-induced astrocyte swelling was accompanied by dynamic changes in AQP4 expression for 48 h. Gunnarson et al. (2008) reported that short-term incubation with glutamate increases astrocyte water permeability via the AQP4 serine 111 residue. However, the dynamic change in AQP4 expression after prolonged incubation with glutamate might involve more complex molecular mechanisms. Meanwhile, we found concentration-dependent increases in AQP4 expression after incubation with 1 or 10 mM glutamate, accompanied by sustained astrocyte swelling. These results confirm that AQP4 plays an important role in the mechanisms underlying astrocyte swelling. Consistent with our findings, the majority of previous studies have found that AQP4 expression in astrocytes is altered in response to various stimuli, including oxygen-glucose deprivation/reoxygenation, hypertonic shock, and P2X7 receptor activation (Lee et al., 2008). Using AQP4 gene silencing, we showed that AQP4 mediates glutamate-induced swelling of cultured astrocytes. Indeed, AQP4 mislocalization or knockdown has convincingly been shown to alleviate brain swelling following ischemia and other brain injuries. For example, Manley et al. (2000) found that mice lacking AQP4 show significantly improved outcomes in models of cellular brain edema, such as those involving cerebral ischemia or water intoxication. Recent studies reported reduced infarct volumes and brain edema in AQP4-deficient mice after tMCAO, and reduced brain swelling and improved neurological outcomes in AQP4 knockout mice following controlled cortical impact brain injury (Yao et al., 2015a,b; Hirt et al., 2017). Similarly, we also found increased AQP4 expression and higher GFAP levels accompanying brain swelling after tMCAO, suggesting that AQP4 plays a crucial role in brain edema. There are two main types of brain edema: cytotoxic and vasogenic. Cytotoxic edema is the predominant type in the early stage of ischemic stroke, and vasogenic edema is usually found in late stroke. AQP4 can inhibit the formation of cytotoxic edema and promote the elimination of vasogenic edema. In fact, over the course of disease development in the setting of mixed edema, the effects of AQP4 are very complex. Our findings show that AQP4 deletion might decrease astrocyte swelling. This could lead to a better understanding of the protective mechanisms of astrocytes associated with cytotoxic edema brain edema and provide more effective approaches to brain edema therapy.

A large number of studies indicate that group I mGluRs are involved in brain injury and astrocyte excitotoxicity (Yuan and Wang, 1996; Vanzulli and Butt, 2015). However, previous studies found no evidence that mGluR5 activation induced AQP4 expression and astrocyte swelling. In this study, we found that mGluR5 activation by an exogenous agonist mimicked the effect of glutamate on cell swelling, and this effect was abolished by co-incubation with an mGluR5 antagonist but not a GLT inhibitor. Moreover, we confirmed increased co-expression of mGluR5 and AQP4 in the pericapillary area and cell membranes of astrocytes after tMCAO, suggesting that ischemic cerebral edema may be related to regulation of AQP4 by mGluR5; more specifically, AQP4 expression may be regulated by mGluR5 to control astrocyte water permeability. As mGluR5 is a G-protein coupled receptor, the mechanisms underlying the effect of mGluR5 activation on AQP4 expression appear to be complex. The majority of research indicates that mGluR5, which couples to G_q/11_, can activate phospholipase C (PLC) β1 and subsequently cause inositol 1,4,5-trisphosphate (IP3) formation and calcium (Ca^2+^) release from intracellular stores (Iacovelli et al., 2013). Stimulation of Group I mGluRs including mGluR1 and 5 can cause increase intracellular Ca^2+^ and initiate Ca^2+^ signaling pathways (Wang et al., 2009). Thrane et al. (2011) found that hypo-osmotic stress initiates astrocytic Ca^2+^ spikes and that deletion of AQP4 reduces these signals. Ca^2+^ signaling is involved in AQP4-mediated astrocytes swelling through regulatory volume decrease (Mola et al., 2016). Gunnarson et al. (2008) showed that the effect of glutamate is Ca^2+^-dependent and may be mediated by the activation of nitric oxide (NO) production and protein kinase G (PKG)-mediated phosphorylation of AQP4 at serine 111. However, other studies have shown that a number of signaling pathways including p38 MAPK, ERK1/2, JNK, PKA, PKC, casein kinase II (CKII), and Ca^2+^/calmodulin-dependent protein kinase II (CaMKII) are involved in regulating AQP4 and mGluR5 expression (Arima et al., 2003; Gunnarson et al., 2004; Nagelhus and Ottersen, 2013; Kritis et al., 2015). Therefore, further studies are needed to explore the regulatory mechanisms underlying the effect of mGluR5 activation on AQP4 expression levels, especially Ca^2+^ signaling pathways. Our experimental results indicate potential roles for AQP4 and mGluR5 in treating conditions associated with brain water balance disturbances.

In conclusion, we have first provided evidence that AQP4 mediated glutamate-induced astrocyte swelling, by using an AQP4 RNAi-based approach, and which appeared to be mediated by activation of mGluR5. Further studies are needed to explore the mechanisms underlying the regulation of mGluR5 on AQP4 expression. Considering that AQP4 may play different roles in alternative phase of the diseases and trigger downstream signaling events participating in the development of disease, the effects of AQP4 on brain edema remain to be explored. Together, these results support the potential therapeutic utility of AQP4 in treating brain edema induced by brain injuries including ischemic stroke.

## Author Contributions

ZS contributed to acquisition, analysis, interpretation of data and to drafting of the manuscript; WZ, YaL, YiL, LX, QF, MW, MJ, YW, LD and XY contributed to acquisition, analysis and interpretation of data for the work and to revision of the manuscript for important intellectual content; SY contributed to the conception of the work and to revision of the manuscript for important intellectual content; FY conceived and designed the experiments for the work and contributed to acquisition, analysis, interpretation of data and to drafting of the manuscript; All authors revised and approved the final version of the manuscript to be published and agreed to be accountable for all aspects of the work.

## Funding

This work was supported by the National Natural Science Foundation of China, No. 81271286 (to FY) and Natural Science Foundation of Beijing, No. 7152027 (to FY).

## Conflict of Interest Statement

The authors declare that the research was conducted in the absence of any commercial or financial relationships that could be construed as a potential conflict of interest.
